# The Elevated Rate of Cesarean Section and Its Contribution to Non-Communicable Chronic Diseases in Latin America: The Growing Involvement of the Microbiota

**DOI:** 10.3389/fped.2017.00192

**Published:** 2017-09-04

**Authors:** Fabien Magne, Alexa Puchi Silva, Bielka Carvajal, Martin Gotteland

**Affiliations:** ^1^Microbiology and Mycology Program, Institute of Biomedical Sciences (ICBM), Faculty of Medicine, University of Chile, Santiago, Chile; ^2^Faculty of Medicine, Andres Bello University, Vina del Mar, Santiago, Chile; ^3^Department of Women and Newborn’s Health Promotion, University of Chile, Santiago, Chile; ^4^Department of Nutrition, Faculty of Medicine, University of Chile, Santiago, Chile; ^5^Institute of Nutrition and Food Technology (INTA), University of Chile, Santiago, Chile

**Keywords:** cesarean section, gut microbiota, childhood diseases, Latin America, metabolic diseases, chronic immune disorders

## Abstract

The current recommendation of the World Health Organization (WHO) regarding cesarean section (C-section) is that this clinical practice should be carried out only under specific conditions, when the health or life of the mother/newborn dyad is threatened, and that its use should not exceed 10–15% of the total deliveries. However, over the last few decades, the frequency of C-section delivery in medium- and high-income countries has rapidly increased worldwide. This review describes the evolution of this procedure in Latin American countries, showing that today more than half of newborns in the region are delivered by C-section. Given that C-section delivery is more expensive than vaginal delivery, its use has increased more rapidly in the private than the public sector; nevertheless, the prevalence of C-section deliveries in the public sector is higher than the WHO’s recommendations and continues to increase, representing a growing challenge for Latin America. Although the medium- and long-term consequences of C-section delivery, as opposed to vaginal delivery, on the infant health are unclear, epidemiological studies suggest that it is associated with higher risk of developing asthma, food allergy, type 1 diabetes, and obesity during infancy. These findings are important, as the incidence of these diseases in the Latin American pediatric population is also increasing, particularly obesity. Although the link between these diseases and delivery mode remains controversial, recent studies indicate that the establishment of the gut microbiota is delayed in infants born by C-section during the postnatal period, i.e., during a critical developmental window for the maturation of the newborn’s immune system. This delay may favor the subsequent development of inflammatory and metabolic disorders during infancy. Accordingly, from a public health perspective, it is important to slow down and eventually reverse the pattern of increased C-section use in the affected populations.

## Introduction

For a long time, maternal mortality associated with pregnancy, birth, and puerperal complications has represented a significant public health problem. One of the strategies aimed at improving mother and newborn outcomes and decreasing mortality in this period is delivery by cesarean section (C-section). This procedure is mainly indicated in the case of placenta praevia, when the fetus is not adequately positioned for birth, when the mother has uncontrolled high blood pressure or diabetes, or in case of multiple pregnancies. However, C-section is not without risk for the mother/infant dyad, and a cautious medical evaluation is required to determine whether the benefits exceed the risks and justify its use (1). A 14-year follow-up of more than two million Canadian women showed that those with low-risk pregnancies undergoing their first C-section were three times more likely to die or suffer serious complications, including infections, blood clots, and heart attack, compared with these who delivered vaginally (2). An additional concern is that mothers who undergo C-section surgery need more time to recover than those who give birth vaginally. Similarly, newborns delivered by C-section have a higher risk of suffering respiratory distress and are less likely to be breastfed than those born vaginally (3). The pioneering studies of Lagercrantz have shown that many of the newborn’s health benefits of vaginal delivery are related to the surge of plasma cortisol and catecholamines that occurs during passage through the birth canal. This hormonal increase positively impacts postnatal glycemia levels, blood pressure, alveolar liquid absorption, and body temperature, and also contributes to neurological adaptation. In contrast, this hormone surge, as well as changes in the physiological parameters previously described, is clearly attenuated in infants born through scheduled C-section (4). In addition to acute negative effects on the newborn, C-section is currently emerging as a risk factor for the development of metabolic and immune diseases, whose incidence is rapidly increasing worldwide. Considering these and other antecedents, the World Health Organization (WHO) currently estimates that C-sections should not exceed 15% of all deliveries (5).

This review describes the rapid increase of this procedure in Latin America and the growing health challenge that it represents for these countries, taking in consideration its frequent association with increased risk of metabolic and immune disorders in the infancy, and the fact that most of these disorders are currently growing in this continent. Finally, we also emphasize the microbiota as a factor that could eventually explain the relationship between C-section and metabolic and immune diseases.

The search of scientific literature was performed using Medline and the Scielo bibliographic databases. Scielo (Scientific Electronic Library Online) is a digital library mainly grouping scientific publications from Latin American countries. We selected studies describing C-section rates in different countries including from Latin America and the impact of C-section on immune and metabolic diseases. Combinations of the following key words were used: “ cesarean section,” “C-section,” “Vaginal Delivery,” “ Latin America,” “South America,” “infant health,” “Type-1 diabetes,” “Celiac disease,” “Allergic diseases,” “asthma,” “Obesity,” “metabolic diseases,” “Type-2 diabetes,” “chronic immune disorders,” “mucosal immune system,” “bacterial gut colonization,” “early gut colonization,” “vaginal seeding,” “prebiotics,” “probiotics,” “microbiome,” and/or “microbiota.” Search was carried out for the articles published during the 1995–2017 period. Full-text articles were downloaded and reviewed to identify those representing the intended context, and data were subsequently extracted from the relevant publications.

## The Uncontrolled Increase of C-Section in Latin America

Despite WHO recommendations, the prevalence of C-section has been increasing worldwide over the last decades, from 6.7% in 1990 to 19.1% in 2014; this increase has been more drastic in middle- and high-income countries than in developing areas (6). A study reported that C-section rate was less than 10% in 54/137 countries, between 10 and 15% in 14 countries and surpassed 15% in 69 countries (7). In the countries with low C-section rates, several factors played a role in keeping these rates low: its higher financial cost, a lack of adequate surgical infrastructure (in rural areas), and misconceptions/lack of information which cause women who are in true need of C-section reluctant to accept the procedure (8). In countries with rates greater than 15%, 6.2 million unnecessary C-sections were performed in 2008, representing a global cost of USD$2.32 billion. While increased use of C-section deliveries has improved rates of maternal and newborn mortality in low-income countries, there are few data in middle- and high-income countries, confirming that the majority of C-sections performed in these countries are unjustified (9, 10).

Currently, South America is the region with the highest C-section rate (42.9%), followed by North America (32.3%), Oceania (31.1%), Europe (25%), and Asia (19.2%) (6). This is not a new trend as in the 1990s, Latin American and the Caribbean countries already had the world’s highest C-section rate (about 23%). This tendency has accelerated in recent years, as South America was the region that saw the largest increase in C-section deliveries from 1990 to 2014 (19.4%). A correlation between country’s *per capita* gross domestic product and C-section rates has been reported for 18 Latin American countries (6). The C-section rate in Uruguay, Chile, Brazil, and Colombia, which together represents more than 40% of births in the region, has increased significantly during the last decade (Table 1). Peru has the lowest C-section rate in the region (~30%); however, it has also seen the greatest increase in recent years. Brazil is the country with the highest C-section rate in the world, reaching 40–45% in the public and 80–95% in the private sector, with high variability according the region/city (11, 12). Interestingly, this country is also ranked among the top 10 with the highest number of preterm births worldwide (13) and some researchers have suggested a link between C-section rate and the rise in premature births (14).

**Table 1:** Changes in C-section rates in Latin American countries.

Country	Year	Rate (%)	Year	Rate (%)	Increase (%)	Reference
Peru	2000	13.0	2014	28.6	120	(15, 16)
Uruguay	2000	23.9	2015	43.1	80	^a^
Chile	1986	27.7	2015	44.7	61	(17, 18)
Brazil	1998	39.2	2013	56.7	45	(19, 20)
Colombia	1998	24.9	2013	45.7	84	(21)
Argentina	1995–2000	25.4	2011	29.1	15	(22)

*^a^Ministerio de Salud de la República Oriental de Uruguay. Estadísticas Vitales. Available from: http://www.msp.gub.uy/EstVitales/*

Unfortunately, it may be considered that C-sections have become as a part of the “modern” maternal lifestyle, a cultural norm, and a marker of economic status in the Latin American countries. This is supported by the fact that the rate of cesarean delivery is twofold higher in Latin American women living in Europe than in the native women (23–25). Although this phenomenon might be explained by linguistic barriers, a recent study showed that the C-section rate in immigrant Brazilian women living in Portugal (and sharing the same language) was about 48%, compared with 35.4% in the Portuguese women. This suggests that cultural background is an important factor determining the selection of the mode of delivery (26). C-section without medical indication is being preferred by a growing number of pregnant women, due to the erroneous perception that it is safer for both the mother and newborn (27), the bad experience of previous delivery, the fear of vaginal delivery and labor-associated pain, and the successfully lobbying of clinics or medical staff. Indeed, for them, C-section is more convenient due to the frequent lack of available hospital beds and because it is plannable and economically more interesting (28–30). However, it is noteworthy that childbirth medicalization can leave women with the feeling that they are no longer active participants in the birth process. In some cases, this may eventually result in disrespectful, abusive or coercive situations affecting mother’s rights (31). This is particularly illustrated by a recent article published by the Daily Telegraph reporting the case of a woman with two previous C-sections, who left the hospital after the doctors denied her a vaginal delivery, and who was subsequently arrested and forced to have her baby by cesarean. Accordingly, it is clear that more emphasis should be placed on initiatives such as the “Maternal and Friendly Delivery Initiative” issued from the Coalition for Improving Maternity Services whose mission is, among others, to promote a full range of options for pregnancy, childbirth and baby feeding, respecting the decisions of mothers (32). Based on these antecedents and with the aim to slow down this “C-section epidemic,” the Brazilian government recently passed a law requiring pregnant women to sign an informed consent form acknowledging the risks of C-section before going into surgery. Simultaneously, it also launched a partnership with several hospitals to promote vaginal birth. A preliminary evaluation suggests a 76% increase in vaginal births, as well as a reduction in C-section associated complications in those hospitals involved in this initiative. However, it is clear that more time is necessary to evaluate the real impact of this new regulation on C-section rate in Brazil.

## C-Sections as a Possible Risk Factor for Infant Health

Experimental data have linked labor avoidance to altered stress responses, immune function, and epigenetic processes in offspring (33–36). A number of epidemiological studies have also been conducted, to determine the eventual impact of birth by C-section on health risks during infancy; here, we summarize these findings.

### Type 1 Diabetes (T1D)

A meta-analysis of 9,939 cases of T1D showed that C-section was associated with a higher risk of this childhood disease (OR: 1.19 [1.04–1.36]) after adjusting for gestational age, birth weight, maternal age, birth order, breastfeeding, and maternal diabetes (37). In an Australian study examining perinatal risk factors for T1D in children under 6 years of age, planned C-section was again shown to increase the risk of T1D (OR: 1.30 [1.01–1.69]) compared to vaginal delivery (38). Similarly, in a Scottish study of more than 320,000 children, the risk of T1D was also higher (adjusted OR: 1.35 [1.05–1.75]) in those delivered by planned C-section than in those born by unscheduled C-section. These children also had higher risk of death (all-causes) prior to 21 years of age (adjusted OR: 1.41 [1.05–1.90]) compared with vaginally born children (39). In contrast, a retrospective study in Denmark looked at the effect of C-section vs. vaginal delivery on chronic immune diseases in all children born from 1973 to 2012, using the Danish national registries. About 1.9 million children were studied and a significantly increased risk of asthma, systemic connective tissue disorders, juvenile arthritis, inflammatory bowel diseases, immune deficiency, and leukemia was observed in those born through C-section; however, they did not find any increased risk of celiac disease, psoriasis, or T1D (40). Similar observations were reported by Clausen et al., also in Denmark (41).

### Celiac Disease

Studies have also suggested an association between C-section and the risk of celiac disease. In a population-based, case–control study of 11,749 Swedish children with biopsy-verified celiac disease and 53,887 age- and sex-matched controls, planned, but not emergency, C-section was associated with an increased risk of later celiac disease (adjusted OR: 1.15 [1.04–1.26]), compared with vaginally delivered children (42). Similarly, a retrospective study using the Danish national registries that included all children born in Denmark between 1997 and 2012 showed that those delivered by unscheduled C-section had an increased risk of ulcerative colitis (adjusted OR: 1.47 [1.02–2.12]) and celiac disease (adjusted OR: 1.52 [1.06–2.20]), whereas children delivered by elective C-section had an increased risk of lower respiratory tract infections and juvenile idiopathic arthritis. The effect of elective C-section was greater than the effect of acute C-section on asthma risk (43).

### Allergic Diseases and Asthma

On the other hand, Bager et al. conducted a meta-analysis of 26 studies (23 cohort and 3 case–control studies) including approximately 1.3 million children, concluding that those born by C-section had higher risk of allergic rhinitis (OR: 1.23 [1.12–1.35]), food allergy or atopy (OR: 1.32 [1.12–1.55]), and asthma (OR: 1.18 [1.05–1.32]) (44). In a study by Black et al., children born by C-section were also more prone to develop asthma requiring hospitalization and to use salbutamol at 5 years of age (adjusted OR: 1.22 [1.11–1.35] and 1.13 [1.01–1.26], respectively), as compared to vaginally delivered children (39). Similar observations have been reported in other studies and meta-analyses (45). Data from 459 children born and cared for in the same maternity ward and followed for 3 years showed that C-section was a significant and independent predictor of food allergy (OR: 3.15 [1.14–8.70]) but not atopic dermatitis (46), confirming the conclusions of a recent systematic review (47). In a retrospective study of more than 7,500,000 Danish children, Kristensen and Henriksen also reported a higher risk of asthma in children born by both unscheduled and elective C-section (OR = 1.06 [1.02–1.10] and 1.24 [1.20–1.28], respectively) (43).

### Obesity

Researchers have also examined the impact of C-section on the subsequent development of obesity. In a Chinese study of 181,380 children, C-section was associated with a higher risk of being overweight after multivariate adjustment [in particular for prepregnancy maternal body mass index (BMI)] (OR: 1.13 [1.08–1.18]) (48). A small, multivariate analysis of 917 American children (~12 years of age) indicated that those born by C-section had approximately twice the likelihood of being obese (OR: 1.87 [1.19–2.95]) or overweight (OR: 1.86 [1.27–2.73]) than those who were born vaginally; this differed by gender, with males at greater risk (49). Recent systematic reviews and meta-analyses have drawn similar conclusions: Li et al. reported an increased risk of overweight/obesity in children (OR: 1.32 [1.15–1.51]), adolescents (OR: 1.24 [1.00–1.54]) and adults (OR: 1.50 [1.02–2.20]) born by C-section, and Kuhle et al. showed an increased risk of obesity after adjustment for maternal BMI (OR: 1.29 [1.16–1.44]) (50, 51). Analysis of mothers who had multiple births found that infants born by vaginal birth following a previous C-section were 31% less likely to become obese than infants born by a repeated C-section delivery. Moreover, within-family analysis showed that individuals born through C-section delivery were 64% more likely to be obese than their siblings born through vaginal delivery. The adjusted risk ratio for obesity among offspring delivered *via* C-section vs. those delivered vaginally was 1.15 [1.06–1.26] (*p* = 0.002). This association between delivery mode and obesity was stronger for children who were born by C-section among women without indications for cesarean delivery (52). These results, therefore, suggest that C-section affects obesity risk, independently of lifestyle and genetic factors.

Collectively, these results indicate that C-section is associated with increased risk of both metabolic diseases and chronic immune disorders during infancy.

## The Growing Incidence of Metabolic Diseases and Chronic Immune Disorders in Latin America

The rapidly increasing frequency of C-section in Latin America is, therefore, an important public health risk that must be considered by both medical professional and public policy makers, as this procedure constitutes a significant risk factor for diseases that are also gradually growing in the region. Latin America has a population of approximately 600 million individuals and is characterized by high ethnic diversity and genetic heterogeneity, due to its Amerindian heritage. Here, we outline recent changes in the incidence of metabolic diseases and chronic immune disorders in Latin America.

### Type 1 Diabetes

Epidemiological data on T1D in Latin America are scarce (53). Higher rates of T1D are observed in countries with higher incomes, increased urbanization, and low Amerindian heritage. For example, a study of children under 14 years of age in Chile reported a lower incidence of T1D in the native aboriginal Mapuche population than in the Caucasian population (0.42 vs. 1.58 per 100,000; *p* < 0.001) (54, 55). Furthermore, a significant increase in the incidence of T1D was observed in the Chilean population from 2000 to 2004 (5.4 vs. 8.33 per 100,000; *p* < 0.04). Similarly, in Mexico a substantial increase (from 3.4 to 6.2 per 100,000) in the incidence of T1D was reported in children under 19 years of age from 2000 to 2010 (56). These studies indicate that the incidence of T1D has increased recently in Latin America.

### Allergic Diseases and Asthma

Data from the International Study of Asthma and Allergies in Childhood showed that the prevalence of asthma or allergic disease ranged from 18 to 27% in Brazil, Uruguay, Paraguay, and Peru; these percentages are similar to those reported in developed countries, which are most affected by this disease (such as Western Europe) (57). However, these incidence rates are growing (+0.32%/year), and are accompanied by mortality rates (primarily due to bronchial asthma) that reach 5.6 per 100,000 in some countries, such as Mexico and Uruguay (57). Asthma is more frequent in urban, low-income populations, mainly due to the accelerated pace of urbanization and migration, the adoption of a westernized lifestyle, and environmental changes (increasing exposure to allergens and indoor/outdoor pollutants). Similar trends have been reported in regards to the incidence of food allergies in Latin America (58, 59).

### Obesity

The prevalence of obesity has increased significantly in Latin America, with about 57% of the adult population classified as overweight and 19% as obese (60, 61). The highest rates of childhood obesity are currently found in Chile (23% in 6-year-olds), and the lowest in Peru (1.8% in children <5 years of age) (62, 63). In Mexico, increased incidence of overweight/obesity has particularly affected adolescent girls and women, with an annual increase of about 1.5% from 1988 to 2012; however, incidence rates have increased much slower for preschoolers (0.3% per year; 1988–2012) and men (0.7% per year; 2000–2012) (64). In Colombia, from year 2005 to 2010, the proportion of individuals classified as overweight increased from 13.9 to 17.5% in children aged 5 to 17 years of age (62). In Brazil, the prevalence of obesity in children and adolescents has risen 240% in the past two decades (65); a similar increase has been observed in Chilean children (66).

While increased C-section rates in Latin America are not solely responsible for the increasing incidence of asthma, food allergies, obesity and T1D in the region, it is nevertheless a contributing factor. Accordingly, decreasing the number of unnecessary C-section surgeries could eventually attenuate the burden of these diseases.

## Why Does C-Section Favor the Development of Metabolic Diseases and Chronic Immune Disorders During Infancy?

### C-Section Alters the Postnatal Establishment of Gut Microbiota in the Newborn

C-section influences the establishment of gut microbiota in the newborns, thus affecting their exposure to bacterial antigens. Bacterial transmission from mother to newborn is currently considered a crucial factor for the establishment of the gut microbiota, which in turn shapes host immune responses (67). In fact, intestinal bacteria are involved in the development of gut-associated lymphoid tissues (GALT), the induction of oral tolerance, and (in rabbits) the diversification of the pre-immune antibody repertoire. In addition, animal studies have shown that an altered (by antimicrobials) gut microbiota early in life affects host physiology, through increased bodyweight and adiposity (68–70). Interestingly, these effects were more pronounced if this alteration occurred prior to weaning, suggesting the presence of a critical period during which an altered gut microbiota can impact the host’s health (68).

It is generally accepted that the fetus grows and develops in a sterile environment within the uterus, and that its passage through the birth canal during delivery is the baby’s first exposure to microorganisms. The pioneering bacterial communities that colonize the intestine originate primarily from the maternal vaginal and fecal microbiota in the case of vaginal delivery and from the mother’s skin microbiota in the case of C-section delivery. In the case of vaginal delivery, this community is primarily composed of facultative anaerobic bacteria, including *Enterococcus, Staphylococcus*, and enterobacteria, which consume the oxygen present in the intestinal lumen, assisting in the creation of an anaerobic environment that strictly anaerobic bacteria can colonize. Accordingly, at the end of the infant’s first week of life, the gut microbiota of a breastfed newborn is dominated by *Bifidobacterium* sp., with lower counts of *Bacteroides* sp., *Clostridium* sp., and lactic acid bacteria. The emergence of this *Bifidobacterium*-dominated microbiota is favored by the significant quantity of oligosaccharides (10–12 g/L) and other bifidogenic compounds (nucleotides, casein-derived glycomacropeptide) contained in breast milk (71, 72). The diversity of the infant gut microbiota increases gradually when solid foods are introduced, eventually reaching a stable adult-like microbiota at 2–3 years of age (73). Various factors influence the gut’s colonization, such as the use of and type of formula, as well as genetic, environmental, and lifestyle factors (73–75).

C-section alters bacterial colonization due to the fact that the fetus has no direct contact with the maternal vaginal/fecal microbiota during delivery, and as antimicrobials are frequently administered during surgery (76). Dominguez-Bello et al. observed that the bacterial composition of meconium samples from vaginally delivered newborns was similar to that of the vaginal microbiota of their mother; in contrast, meconium from newborns delivered by C-section was most similar to the maternal skin microbiota (dominantly composed of *Streptococcus* spp., *Staphylococcus* spp., and *Propionibacterium* spp.) (77). In addition, delivery mode has also been shown to influence the bacterial composition of breast milk, which is another important early bacterial source (78, 79). C-section delivery delays gut colonization by *Bifidobacterium, Lactobacillus*, and perhaps most importantly *Bacteroides* spp. Establishment of these bacteria is thus delayed, and typically mirrors that of a vaginally born infant only at around 6 months of age (80); however, some studies report that residual dysbiosis may persist for longer, until 2 or even 7 years of age (Table 2) (81, 82).

**Table 2:** Long-term alteration of the gut microbiota associated with cesarean section.

Country	Age	Effective	Method	Bacterial diversity difference	Microbial changes	Reference
Finland	6 months	CD = 30 VD = 34	Culture	ND	The colonization rate of *Lactobacillus*-like bacteria was lower in the CD group than in the VD group In the CD group, the colonization rate of the *Bacteroides fragilis* group was only half that of infants in the VD group (36 vs. 76%)	(80)
India	3 months	CD = 10 VD = 73	qPCR	ND	The *Bacteroides*–*Prevotella* group was more abundant in the feces of VD infants compared with those of CD from birth up to three months	(83)
Korea	6 months	CD = 3 VD = 3	16S rDNA pyrosequencing	No	The *Clostridium* genus increased with age in the infants in our study born by CD, while consistently lower levels were observed in infants born by VD during the first 6 months of life	(84)
USA	6 weeks	CD = 12 VD = 11	16S rDNA pyrosequencing Metagenomic	ND	Bacteroidetes were more abundant through 6 weeks of age in stool of VD infants. Infant fecal microbiota in both delivery groups did not resemble maternal rectal or vaginal microbiota. Differences in fecal bacterial gene between CS and VD at 6 weeks clustered in metabolic pathways and were mediated by the abundance of Proteobacteria and Bacteroidetes	(85)
Sweden	24 months	CD = 9 VD = 15	16S rDNA pyrosequencing	Infants born through CS had lower total microbiota diversity	Infants born by CS had a lower abundance and diversity of the Bacteroidetes phylum and were less often colonized with the Bacteroidetes phylum	(82)
Canada	4 months	CD = 6 VD = 18	16S rDNA pyrosequencing	Infants born by elective CS had the lowest richness and diversity	*Escherichia*–*Shigella* and *Bacteroides* species were underrepresented in infants born by CD. Infants born by elective CD had particularly low bacterial richness and diversity	(86)
Finland	7 years	CD = 31 VD = 29	Fluorescent *in situ* hybridization method	ND	Compared with infant born by CD, higher numbers of clostridia were found in children born by VD	(81)

*CD, cesarean delivery; VD, vaginal delivery; ND, not determined*.

Although many studies have reported gut dysbiosis in patients with asthma, allergies, obesity or T1D (87), few studies have examined alterations in the postnatal microbiota of infants that go on to develop one of these pathologies, and those that exist mostly focus on allergies (Table 3). Various microorganisms that colonize the infant gut early in life, such as *Bifidobacterium* (88–91), *Lactobacillus* (90, 91), *Enterobacteriaceae*, and *Clostridium* (92), have been associated with the subsequent development of allergies. Although no specific microorganism has been clearly identified as either protective or inductive, various studies point to *Clostridium difficile* as favoring allergy development. In fact, colonization of the gut by this opportunistic pathogen is accompanied by disruption to the microbiota prior to the onset of allergic disease. As previously stated, various studies have described lower microbial diversity in children who subsequently developed allergies. One study showed that atopic eczema was preceded by decreased microbial diversity, specifically affecting the genus *Bacteroides* (93). It is possible that a more diverse gut microbiota is more efficient in improving the maturation of the immune system and reducing the risk of chronic immune disorders (94).

**Table 3:** Diseases associated with gut microbiota alterations in early life.

Disease	Country	Age of diagnosed disease (years)	Effective *n* (control group)	Early bacterial diversity	Early gut microbiota data	Age of microbiome analysis	Method	Reference
Allergy	Sweden	5 years	*n* = 13 (*n* = 25)	ND	↓ Prevalence of lactobacilli group I (*L. rhamnosus, L. casei*, and *L. paracasei*) at the first week ↓ Prevalence of *Bifidobacterium adolescents* at the first week. ↓ Prevalence of *Clostridium difficile* during first 2 months.	1 week 1 month 2 months	qPCR	(90)
Allergy (against food and inhalant allergens)	Sweden	5 years	*n* = 16 (*n* = 19)	ND	↓ Lactobacilli prevalence at first weeks of life ↓ *Bifidobacterium bifidum* prevalence at 1 week of age	1 week 2 weeks 1 month 2 months 12 months	qPCR	(91)
Allergy (atopic dermatitis and allergic rhinitis/rhinoconjunctivitis)	Estonia Sweden	2 years	*n* = 18 (*n* = 26)	ND	↑ Clostridia prevalence at 3 months ↓ *Bacteroides* prevalence at 12 months	1 week 1 month 3 months 6 months 12 months	Culture methods	(88)
Asthma	Sweden	7 years	*n* = 8 (*n* = 39)	↓ Total bacteria	NS	1 week 1 month	16S rDNA pyrosequencing	(95)
Asthma	Netherlands	6–7 years	*n* = 99 (*n* = 832)	ND	↑ *C. difficile* prevalence	1 month	qPCR	(96)
Atopic eczema	Sweden	2 years	*n* = 20 (*n* = 20)	↓ Total bacteria ↓ Phylum Bacteroidetes ↓ Genus Bacteroides	NS	1 month	16S rDNA pyrosequencing	(93)
Atopic eczema	Sweden Great Britain Italy	18 months	*n* = 15 (*n* = 20)	↓ Total bacteria	ND	1 week	T-RFLP TTGE	(97)
Atopic sensitization	Finland	12 months	*n* = 12 (*n* = 17)	ND	↑ Clostridia concentration ↓ *Bifidobacterium* concentration	3 weeks	FISH	(89)
Eczema	US	6 months	*n* = 9 (*n* = 12)	↓ Total bacteria	ND	1 month 4 months	DGGE	(98)
Eczema	Australia	12 months	*n* = 33 (*n* = 65)	↓ Total bacteria	ND	1 week	T-RFLP	(99)
Eczema	Singapore	2 years	*n* = 28 (*n* = 32)	ND	↑ Enterobacteriaceae abundance ↑ Clostridium perfringens abundance	3 days 1 month 3 months 1 year	FISH-FC	(92)
Eczema	Singapore	5 years	*n* = 15 (*n* = 19)	ND	↓ *Bifidobacterium* abundance	3 days 1 month 3 months 1 year	FISH-FC	(92)
Eczema	Netherlands	2 years	*n* = 311 (*n* = 646); all samples have not been analyzed	ND	↑ Prevalence and concentration of *Escherichia coli* ↑ *C. difficile* prevalence	1 month	qPCR	(100)
Obesity	Finland	7 years	*n* = 25 (*n* = 24)	ND	↓ *Bifidobacterium* concentration ↑ *Staphylococcus aureus* concentration	6 months 12 months	FISH-FC qPCR	(101)

*NS: no significant differences in relative abundance of bacterial phyla, classes, and genera between children with or without the disease*.

*ND, not determined; ↑, increasing (compared to control group); ↓, decreasing (compared to control group); T-RFLP, terminal restriction fragment length polymorphism; TTGE, temporal temperature gradient gel electrophoresis; DGGE, denaturating gradient gel electrophoresis; FISH, fluorescence *in situ* hybridization; FISH-FC, fluorescence *in situ* hybridization-flow cytometry*.

A longitudinal study analyzing the gut microbiota of infants from birth to 7 years of age, showed that those who became overweight at 7 years harbored significantly fewer *Bifidobacterium* sp. at 6 and 12 months of age compared with children who were normal weight at 7 years of age (101). More longitudinal studies evaluating the development of the early gut microbiota and its impact on the development of obesity, T1D, and food allergy are necessary to confirm whether alterations in the microbiota’s composition and/or a delay in the colonization process precedes the development of these diseases.

### Postnatal Gut Dysbiosis Affects the Maturation of the Infant’s Mucosal Immune System

Decreased levels of intestinal *Bacteroides* spp. associated with C-section delivery are significant, as this microorganism strongly influences host physiology and metabolism. Studies in germ-free mice indicate that *Bacteroides thetaiotaomicron* participates in maintaining gut barrier function by increasing epithelial expression of the small proline-rich protein 2A, a polymeric immunoglobulin receptor, and matrilysin, a matrix metalloproteinase involved in defensin activation in Paneth cells (102, 103). The combination of *Bacteroides fragilis* and *Bacillus subtilis* consistently promoted GALT maturation in the rabbit appendix and led to the development of the pre-immune antibody repertoire, as reflected by increased somatic diversification of VDJ-Cμ genes in appendix B cells (104). *Bacteroides* species have also been implicated in the improvement of gut immunity and inflammatory response, by stimulating the plasmocyte production of secretory IgA (105). A polysaccharide A-expressing strain of *B. fragilis* also suppressed the release of the pro-inflammatory cytokine IL-17 in the intestine (106). Interestingly, adult mice born by C-section showed a lower proportion of Foxp3^+^ regulatory T cells and tolerogenic CD103^+^ dendritic cells, along with lower IL-10 expression in mesenteric lymph nodes and the spleen, suggesting negative long-term systemic effects of C-section delivery on the immune system of these animals (107).

Collectively, these results suggest that high levels of *Bacteroides* sp. (and eventually *Bifidobacterium*) present in the infant gut during the first 6 months following vaginal delivery exert health-promoting effects by favoring the maturation of GALT, mucosal immunity and oral tolerance, and reducing the risk of immune and metabolic disorders during infancy. On the contrary, for infants born by C-section delivery, the absence (or low abundance) of these microorganisms during this important physiological window of development appears to result in disturbances of immune homeostasis and a higher risk of developing metabolic and immune disorders later in life.

## Interventions That may Improve Gut Colonization in Infants Born by C-Section

With the aim of introducing the appropriate maternal microbiota to an infant delivered *via* C-section, and to decrease the risk of immune and metabolic disorders, Dominguez-Bello et al. carried out a study in which they immediately swabbed newborns after delivery with a gauze previously set in the maternal vagina for 1 h prior to surgery (108). The gut bacterial community of these newborns was enriched in vaginal bacteria during the first 30 days of their life, similar to vaginally delivered children; in children born by C-section who were not swabbed, these bacterial populations were underrepresented. Although the long-term health consequences of restoring the microbiota of infants delivered by C-section remain unclear, these results demonstrate that vaginal microbes can be partially restored at birth in C-section-delivered babies (108). One argument against such “vaginal seeding” is the potential presence of pathogens in the maternal vaginal microbiota to be introduced to the newborn.

Another approach proposed to assist in the “normal” colonization of the gut microbiota in infants born by C-section has been the administration of probiotics. Garcia Rodenas et al. observed that the administration formula containing *L. reuteri* DSM17938 did not affect the microbiota of vaginally delivered infants, but promoted fast recovery from gut dysbiosis induced by C-section delivery (109).

## Conclusion

The prevalence of C-section is increasing worldwide, despite WHO recommendations to the contrary, being this increase most acute in Latin America. This phenomenon may be due to several factors, such as: low maternal self-efficacy; a fear of childbirth, which is related to the social construction of this event; fear of labor pain; and a predisposition by the medical team to perform the surgery (110–112). It is therefore important to implement public health programs focused on reversing this trend, being C-section is now considered as a significant risk factor for a several number of immune and metabolic diseases, whose incidences are currently growing worldwide. To reduce unnecessary C-sections and encourage vaginal birth, various strategies should be considered, such as the implementation of standardized protocols, request of a second medical opinion prior to surgery, improving maternal empowerment during pregnancy and delivery, maternal and medical collaboration on birth plans, alternatives to endure labor pain, creation of pleasurable labor environments, allowing the presence of a companion during birth, and midwife-led care during pregnancy and labor (23, 113, 114). Furthermore, as breastfeeding also plays a key role in the establishment of the early gut microbiota following birth, the following strategies should also be considered: adequate support by trained midwifes/lactation experts, public health campaigns supporting/encouraging breastfeeding, restriction on formula hand-out at maternities, and the fact that infants stay in their mother’s hospital room overnight rather than in a separate nursery.

The negative impacts of C-section on later health may be due to the delay in the postnatal establishment of the gut microbiota and subsequent alterations to the maturation of the mucosal immune system. Accordingly, the infant gut microbiota may be a potential target for preventing or attenuating the disorders associated with C-section delivery. However, most studies on the infant gut microbiota and its health impacts cited in this review have been conducted in developed countries. This is a significant bias on our understanding of these phenomena, as the microbiota is highly variable between individuals according their dietary habits, lifestyle, and genetic background (75). Longitudinal studies are therefore necessary to increase our knowledge on the impact of C-section delivery on the development of the gut microbiota during infancy and the incidence of chronic immune and metabolic disorders in developing countries, including Latin America.

## Author Contributions

All authors participated equally in the writing of this review. BC performed the literature review of C-section rates in Latin America. APS performed the literature review of metabolic and immune diseases in Latin America. FM and MG conceived of the idea, the review, and edited this work.

## Conflict of Interest Statement

The authors declare that the research was conducted in the absence of any commercial or financial relationships that could be construed as a potential conflict of interest.
